# Complete mitochondrial genome of *Vanmanenia hainanensis* (Cypriniformes: Gastromyzontidae)

**DOI:** 10.1080/23802359.2021.1927215

**Published:** 2021-05-13

**Authors:** Xingwei Cai, Shuqing Deng, Zhixin Shen

**Affiliations:** a Hainan Academy of Ocean and Fisheries Sciences, Haikou, Hainan Province, China; b Institute of Hydrobiology, Chinese Academy of Sciences, Wuhan, Hubei Province, China; c University of Chinese Academy of Sciences, Beijing, China; d The Changjiang Civilization Museum (Wuhan Natural History Museum), Wuhan, Hubei Province, China

**Keywords:** *Vanmanenia hainanensis*, mitochondrial genome, phylogenetic relationship

## Abstract

*Vanmanenia hainanensis* Chen & Zheng 1980 is endemic to Hainan Island, China. The complete mitogenome of the species was sequenced in this study. It was 16,555 bp in length, containing 13 protein-coding genes (PCGs), 22 tRNA genes, 2 rRNA genes, and 1 control region. The base composition was 29.5% A, 25.4% T, 16.7% G, and 28.4% C. All genes were encoded on the H-strand except for ND6 and 8 tRNA genes, located on the l-strand. Phylogenetic analysis based on 13 protein-coding genes indicated that the genus *Vanmanenia* did not form monophyly and it had the closest relationship with *Formosania*. This study aimed at providing useful genetic information for future studies on taxonomy, phylogeny, and evolution of *Vanmanenia* species.

The genus *Vanmanenia* (Cypriniformes: Gastromyzontidae), a loach group inhabiting fast-flowing streams, is distributed in China, Vietnam and Laos (Chen and Tang 2000). At present, 16 valid species have been found in China (Chen and Tang 2000; Li et al. 2019; Deng and Zhang 2020). *Vanmanenia hainanensis* Chen & Zheng 1980 is endemic to Hainan Island (Zheng and Chen 1980). However, only three complete mitochondrial genomes of *Vanmanenia* species were reported in previous studies (Liang et al. 2014; Chen and Li 2016; Shi et al. 2018). In the present study, we sequenced the complete mitochondrial genome of *V. hainanensis*, which will provide important information for future studies on taxonomy, phylogeny, and evolution of *Vanmanenia* species. The specimen was collected from Changhua River (N 18°53′48.48″, E 109°20′14.31″), Hainan Province, China, in April 2020, and stored in the Museum of Aquatic Organisms at the Institute of Hydrobiology (IHB), Chinese Academy of Sciences (Voucher specimen: IHB2020040086).

Whole mitochondrial genome sequence of *V. hainanensis* had a circular structure of 16,555 bp (GenBank accession number: MW289207). It contained 13 protein-coding genes (ATP6, ATP8, COI–III, Cytb, ND1–6, ND4L), 22 tRNA genes, 2 rRNA genes (12S and 16S rRNA), and 1 control region (d-Loop). The base composition was 29.5% of A, 16.7% of G, 25.4% of T, 28.4% of C, and A + T-biased (54.9%). All mitochondrial genes were encoded on the H-strand except the ND6 gene and 8 tRNA genes, located on the l-strand. Five overlapping regions were observed with 2–10 bp in length. There were 13 intergenic sequences, varying from 1 to 31 bp in length. The total length of the PCGs (13 protein-coding genes) was 11,419 bp, and these genes encode 3797 amino acids. The 22 tRNA genes had lengths ranging from 66 bp (tRNA^Cys^) to 70 bp (tRNA^Lys^). And the 12S, 16S rRNA, and d-loop genes were 953, 1679, and 893 bp, respectively.

The concatenated protein-coding gene sequences were extracted by PhyloSuite (Zhang et al. 2020) from the newly sequenced and other 15 currently available species of Gastromyzontidae. *Lepturichthys fimbriata* and *Jinshaia sinensis* were chosen as the outgroup. The phylogenetic trees of the family Gastromyzontidae were reconstructed using Bayesian inference (BI) and Maximum likelihood (ML) methods by MrBayes (Ronquist et al. 2012) and IQ-TREE (Nguyen et al. 2015) based on 13 PCGs. The genetic distances (p-distance with 1000 bootstraps) were calculated by MEGA 7.0 (Kumar et al. 2016). The two analysis methods (ML and BI) showed an identical topology (Figure 1). The result revealed that the genus *Vanmanenia* did not form monophyly and it clustered with *Formosania* into a well-supported linage, which was also reported in other studies (Zhang, 2012; Chen and Li 2016; Shi et al. 2018; Wan et al. 2019). *Vanmanenia hainanensis* had the closest relationship with *V. maculata*, a species of the same genus. The nucleotide sequence divergence of 13 protein-coding genes between the two species was 8.2%, larger than the intraspecific distance of *V. hainanensis* (3.8%).

**Figure 1.**
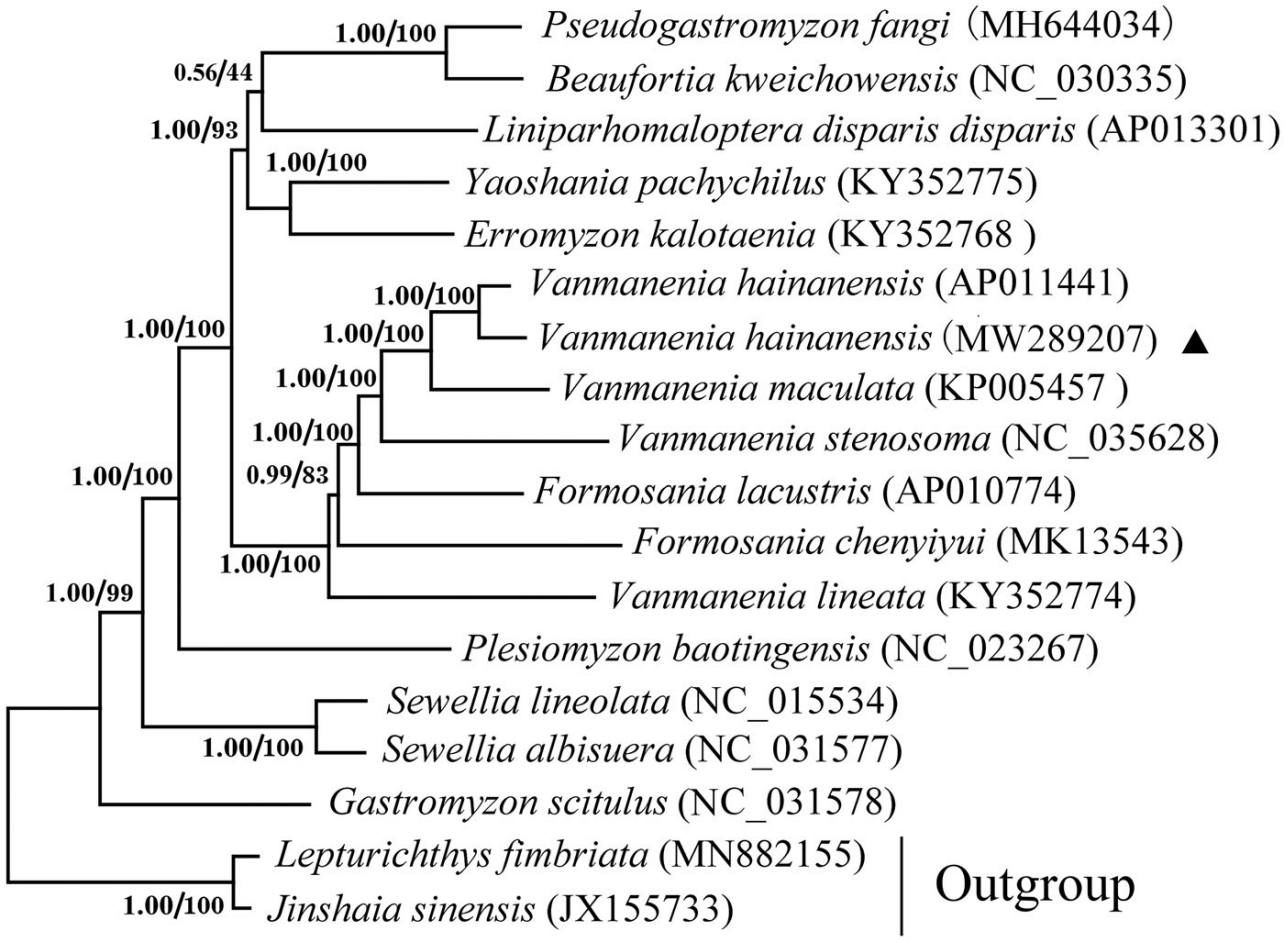
Phylogenetic tree of family Gastromyzontidae reconstructed using Bayesian method (BI) and maximum-likelihood (ML) based on the concatenated dataset of 13 protein-coding genes. Values at the nodes correspond to the support values for BI/ML methods. The GenBank accession number of each species was shown in brackets to the right of the name. Solid triangle indicates the newly sequenced mitogenome.

## Data Availability

The data that support the findings of this study are openly available in GenBank at https://www.ncbi.nlm.nih.gov/nuccore/MW289207, GenBank accession no. MW289207.
